# Tailor-Made Tapering Grafts for Large-Neck Aorta

**DOI:** 10.3400/avd.hdi.21-00117

**Published:** 2022-03-25

**Authors:** Takuro Shirasu, Masaru Kimura, Takanori Kaneko, Takatoshi Furuya, Kaito Fukuda, Motoki Nagai, Yukihiro Nomura

**Affiliations:** 1 Department of Surgery, Asahi General Hospital, Asahi, Chiba, Japan; 2 Department of Surgery, University of Virginia, Charlottesville, VA, USA

**Keywords:** abdominal aortic aneurysm, large neck, hostile neck, open aneurysm repair, aortomegaly

## Abstract

Patients having a large aortic neck poses a challenge in abdominal aortic aneurysm surgery both in endovascular and open aneurysm repair, sometimes necessitating paravisceral or thoracoabdominal aneurysm repair which carries considerable perioperative risk. Here, we describe techniques of using a tailor-made tapering graft in open surgery that can be adjusted for large neck morphology. This technique helps avoid discrepancies between the proximal aorta and graft, and postoperative acute kidney injury by clamping at lower levels. The conscientious use of this technique in selected patients realizes satisfactory outcomes both in the short term and midterm in the demanding anatomy of large aortic necks.

## Introduction

A large aortic neck is one of the hindrances to endovascular aneurysm repair; if it is operated on endovascularly, the risk of type Ia endoleak and subsequent death due to rupture could carry over even five years later in various major devices.^1–3)^ The aneurysms sometimes constrain paravisceral aneurysm repair, which is more invasive with substantial morbidity and mortality both in endovascular and open repair.^4)^ Open repair for a large-neck abdominal aortic aneurysm (AAA) is more durable but not straightforward due to discrepancies in sizes between the proximal aorta and general prosthetic graft. We use tailor-made tapering grafts to address the predicaments when dealing with large proximal aortas.

## Case Report

**Figure 1:**
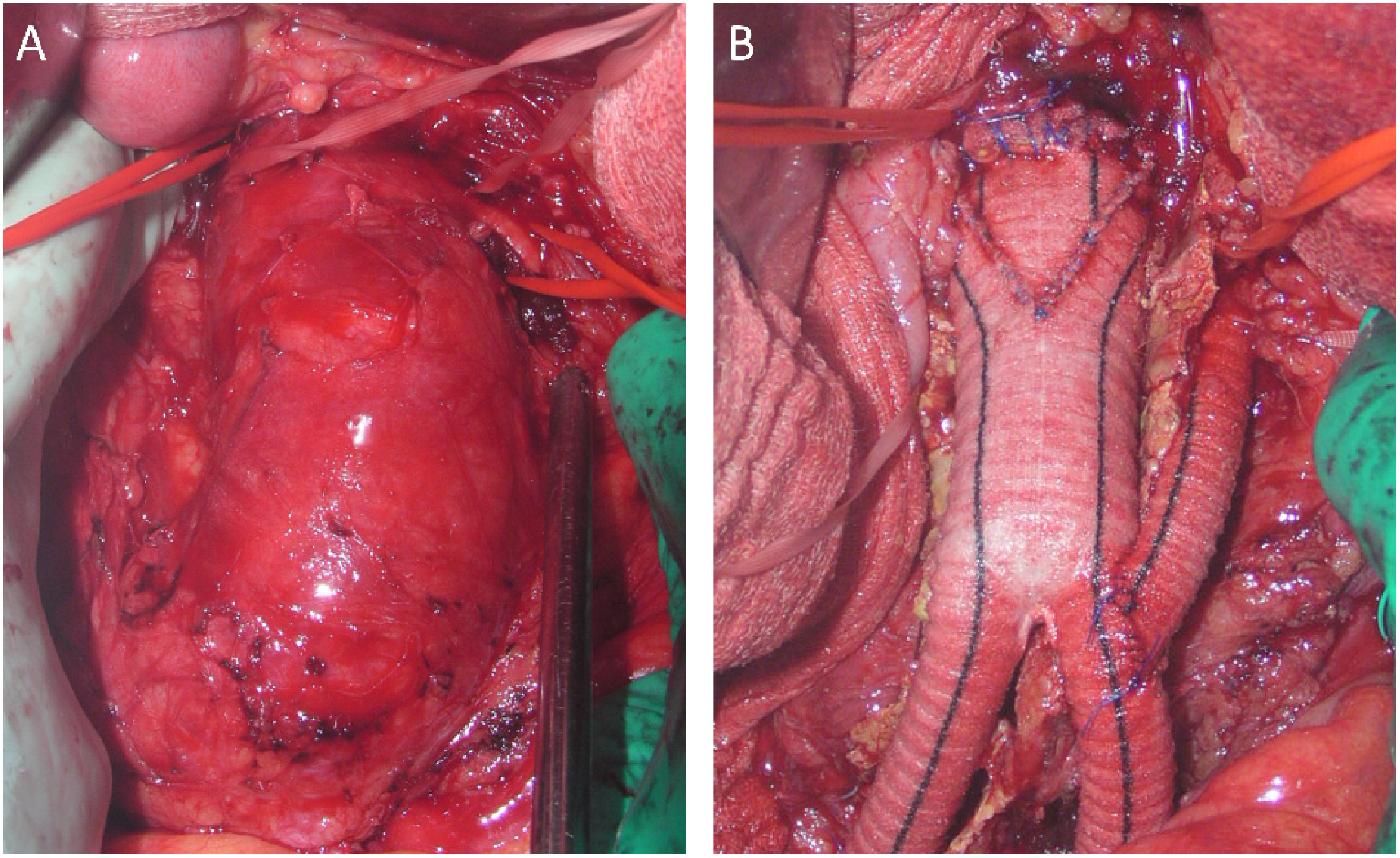
Fig. 1 Preparation of the proximal neck. (**A**) The left renal vein was dissected. (**B**) Both renal arteries and the inter-renal aorta were looped after the division of the left renal vein. The level and size of the proximal aorta were carefully inspected before anastomosis.

**Figure 2:**
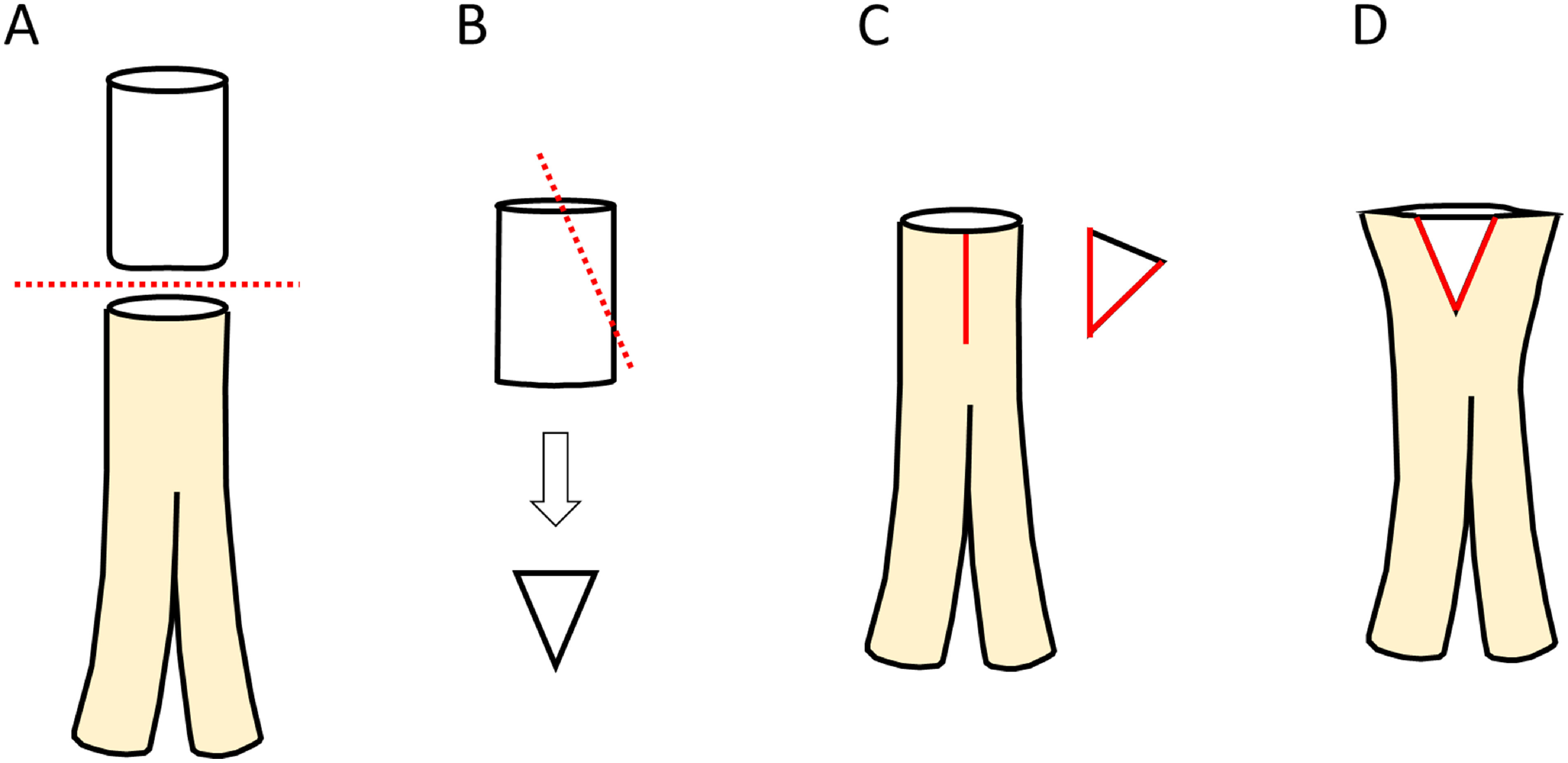
Fig. 2 Schematic of a tailor-made tapering graft. (**A**) Transection of the trunk of the bifurcated graft. (**B**) An isosceles triangle is cut from the detached body of the graft. An acute rather than obtuse isosceles triangle is preferred. (**C**) The longitudinal cut of the forefront of the trunk of the bifurcated graft—note that the length of the incision is the same as the equal sides of the isosceles triangle. (**D**) The isosceles triangle was sutured to the trunk with a 3-0 monofilament polypropylene suture.

**Figure 3:**
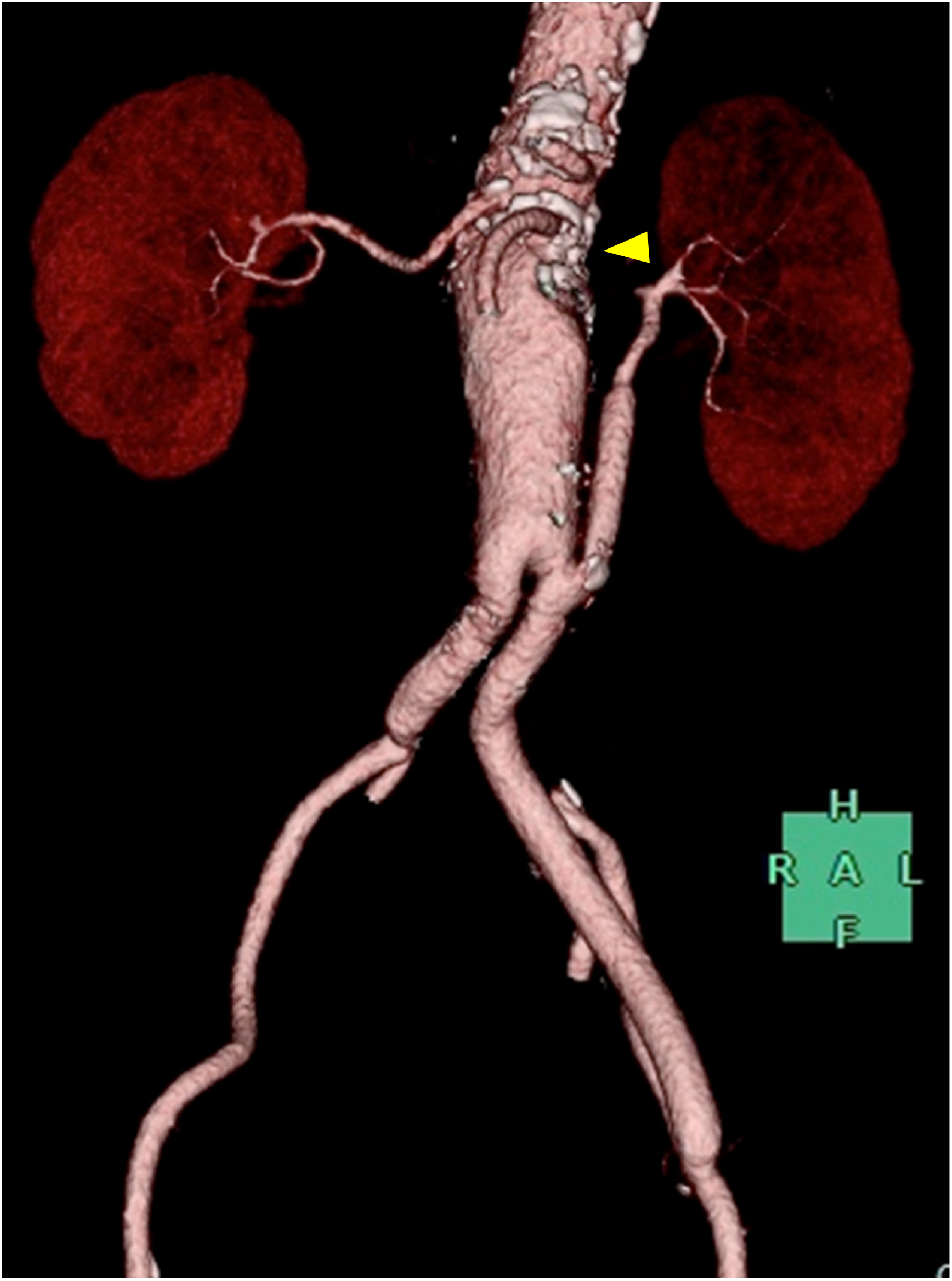
Fig. 3 Postoperative imaging of a tailor-made tapering graft. Contrast-enhanced computed tomography 1 year after surgery. The tailor-made tapering graft was created from Hemashield Gold 18 mm ×9 mm. An arrowhead shows the proximal anastomosis. Note that there is no graft-related complication or thrombus, and both graft limbs were anastomosed to the normal-sized iliac arteries.

An 82-year-old male patient was diagnosed with pararenal AAA of 56 mm in a maximal diameter, that had gradually grown in the 10-year follow-up. The aortic diameters at celiac, superior mesenteric/right renal, and left renal artery levels were 33 mm, 32 mm, and 43 mm, respectively. He chose to undergo open aneurysm repair with informed consent. His aorta was large (aortomegaly); therefore, we planned to clamp it just below the superior mesenteric/right renal artery level (32 mm) and reconstruct the left renal artery with bypass grafting. Through a midline incision, the left renal vein was divided, and the bilateral renal arteries and proximal aorta were looped for clamping (**Fig. 1A**). After confirming the diameter at the anastomosis, a tapering graft was tailor-made on the back table. Using a transected portion of Hemashield Gold (Maquet, Rastatt, Germany), 18 mm×9 mm (**Fig. 2A**), we created an acute isosceles triangle (**Fig. 2B**). The forefront of the trunk of the bifurcated graft was cut longitudinally of the same length as the equal side of the isosceles triangle (**Fig. 2C**). Each vertex of the triangle together with the part on the trunk of the main graft was held with curved Kelly forceps. Two separate running sutures were performed with 3-0 Surgipro™ (Covidien, Dublin, Ireland) for each edge, beginning from the acute vertex (**Fig. 2D**). After clamping, the left kidney was irrigated with cold Ringer’s solutions. Reconstruction was performed using the tailor-made tapering bifurcated graft and left renal artery bypass. The proximal anastomosis was reinforced by an expanded polytetrafluoroethylene felt (**Fig. 1B**). The operation duration was 496 min, and the estimated blood loss was 1725 mL. His postoperative course was uneventful, with the highest serum creatinine levels at 1.40 mg/dL on Day 1. He was discharged on Day 6 and returned to his normal activities. Contrast-enhanced computed tomography taken 1 year after surgery indicated no problems at the site of anastomosis or the tapering graft (**Fig. 3**). At 3 years, he neither had renal impairment (serum creatinine level 0.79 mg/dL) nor anastomotic degeneration. He gave written informed consent for case presentation.

## Discussion

A large aortic neck is not rare in patients with AAA. In real-world registries, proximal aortic diameters >25 mm and >30 mm were observed in 37.6% and 7.7% of patients/the population, respectively, both of which lead to higher risks of delayed type Ia endoleak after conventional endovascular aneurysm repair.^1,2)^ Considering the natural enlargement of ectatic aortas (26–29 mm) together with the factors of radial force and oversizing of stent grafts, this observation is legitimate, especially in the long term.^5,6)^ Therefore, open repair is a reasonable option for good-risk patients having AAA combined with a large neck.

The tailor-made tapering graft presented in the current report can be applied to any diameter at the anastomosis. Tips should include an adequate length of the trunk because an acute rather than obtuse isosceles triangle makes for a natural taper (**Figs. 2B** and **2D**). We have employed this method for five other patients with large proximal aortas (aortic diameters at anastomosis ranging from 30 to 35 mm). We preferred using expanded polytetrafluoroethylene felts at the anastomoses. Compared to other pararenal open repairs with conventional grafts (n=22), tailor-made tapering grafts treated significantly larger aortas (3.2±0.27 cm vs. 2.4±0.47 cm, p=.0007) and yet contributed to withholding renal ischemia (0 min vs. 42±27 min, p=.002) and showing better trends in operation time (257±33 min vs. 307±69 min, p=.13), estimated blood loss (515±173 mL vs. 806±393 mL, p=.12), and postoperative acute kidney injury (0% vs. 36%, p=.28). During a mean nine-year follow-up, no graft-related problems were observed. One patient with an anastomotic dilatation (42 mm) had been observed non-operatively without any event. Our results were further supported by others’ observations wherein reconstruction at the level just below the renal arteries performed better in the short term without increasing anastomotic degeneration at 3 years.^7)^

**Figure 4:**
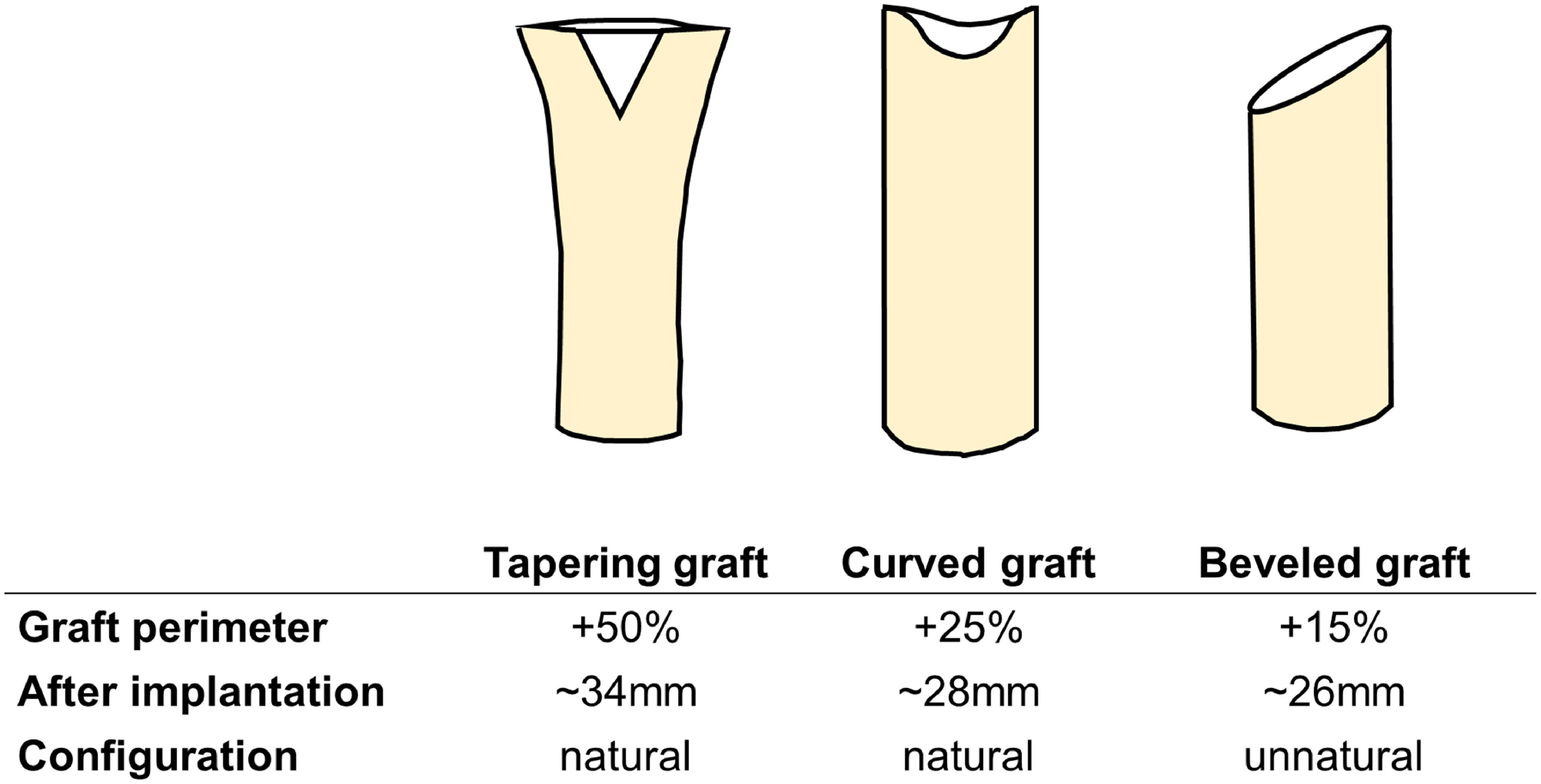
Fig. 4 Characteristics of different types of grafts used in large-neck aortas. The size after implantation takes +25% growth in size into consideration. The tapering graft handles the largest diameter range with natural configuration.

As described in the literature, there are two distinct types of arterial enlargements without reaching aneurysms: general arteriomegaly and local arteriomegaly (or aortomegaly).^8)^ Commercially available larger-size grafts (e.g., 24 mm×12 mm or 22 mm×11 mm) may be used for the former cases. However, there are a few techniques available in the case of a large-neck aorta with a huge discrepancy in size from the distal arteries—composite grafts made of different sizes of prosthetic grafts or beveled grafts. Although we applied the inclusion technique for proximal anastomosis, any size discrepancy should be avoided as much as possible because it can lead to either leakage or wrinkles, which could potentially cause luminal narrowing of the renal arteries. Tailor-made tapering grafts can increase the graft diameter by up to 50% in ex-vivo experiments (**Fig. 4**). We also conform to grafts with a curved edge, which is estimated to increase the perimeter by up to 25%. However, widely used beveled grafts (e.g., 30 degrees) will increase the perimeter by up to 15%. Supposing Dacron grafts expand by 25% when implanted; 18 mm grafts could accommodate up to 34 mm, 28 mm, and 26 mm aortas in tapering, curved, and beveled grafts, respectively. Considering the contemporary concern regarding adequate experience and performance level among young vascular surgeons,^9–11)^ open surgical techniques ensuring safe and effective outcomes are highly desirable.

## Conclusion

The advantages of our tapering grafts lie in their flexibility to handle the widest ranges of aortic diameters and natural configurations without crushing the orifices of the renal arteries. Anastomosis at aneurysms should be avoided. In selected cases with a large-neck aorta such as aortomegaly, tailor-made tapering grafts contribute to decent outcomes both in the short term and midterm.
